# Mechanotransduction in subchondral bone microenvironment and targeted interventions for osteoarthritis

**DOI:** 10.1016/j.mbm.2024.100043

**Published:** 2024-02-05

**Authors:** Rui Feng, Wenhui Hu, Yuheng Li, Xuan Yao, Jianmei Li, Xiaoming Li, Jing Zhang, Yu Wu, Fei Kang, Shiwu Dong

**Affiliations:** a Department of Biomedical Materials Science, College of Biomedical Engineering, Army Medical University (Third Military Medical University), Chongqing 400038, PR China; b College of Bioengineering, Chongqing University, Chongqing 400044, PR China; c State Key Laboratory of Trauma and Chemical Poisoning, Army Medical University (Third Military Medical University), Chongqing 400038, PR China

**Keywords:** Mechano-signaling, Osteoarthritis, Subchondral bone microenvironment, Sensation and respondence, Targeted interventions

## Abstract

Osteoarthritis (OA) is a progressive degenerative joint sickness related with mechanics, obesity, ageing, *etc*., mainly characterized by cartilage degeneration, subchondral bone damage and synovium inflammation. Coordinated mechanical absorption and conduction of the joint play significant roles in the prevalence and development of OA. Subchondral bone is generally considered a load-burdening tissue where mechanosensitive cells are resident, including osteocytes, osteoblast lineage cells, and osteoclast lineage cells (especially less concerned in mechanical studies). Mechano-signaling imbalances affect complicated cellular events and disorders of subchondral bone homeostasis. This paper will focus on the significance of mechanical force as the pathogenesis, involvement of various mechanical force patterns in mechanosensitive cells, and mechanobiology research of loading devices *in vitro* and *in vivo*, which are further discussed. Additionally, various mechanosensing structures (*e.g*., transient receptor potential channels, gap junctions, primary cilia, podosome-associated complexes, extracellular vesicles) and mechanotransduction signaling pathways (*e.g*., Ca^2+^ signaling, Wnt/β-catenin, RhoA GTPase, focal adhesion kinase, cotranscriptional activators YAP/TAZ) in mechanosensitive bone cells. Finally, we highlight potential targets for improving mechanoprotection in the treatment of OA. These advances furnish an integration of mechanical regulation of subchondral bone homeostasis, as well as OA therapeutic approaches by modulating mechanical homeostasis.

## Highlights

• Mechanical force is one of the significant pathogenesis of osteoarthritis.
• Various mechanical force patterns involve in subchondral bone cells.
• Mechanosensing structures and mechanotransduction signaling pathways mediate mechanical conduction in subchondral bone cells.
• Targeting mechanical homeostasis of subchondral bone facilitates the treatment for osteoarthritis.

## 1 Introduction

**Fig. 1:**
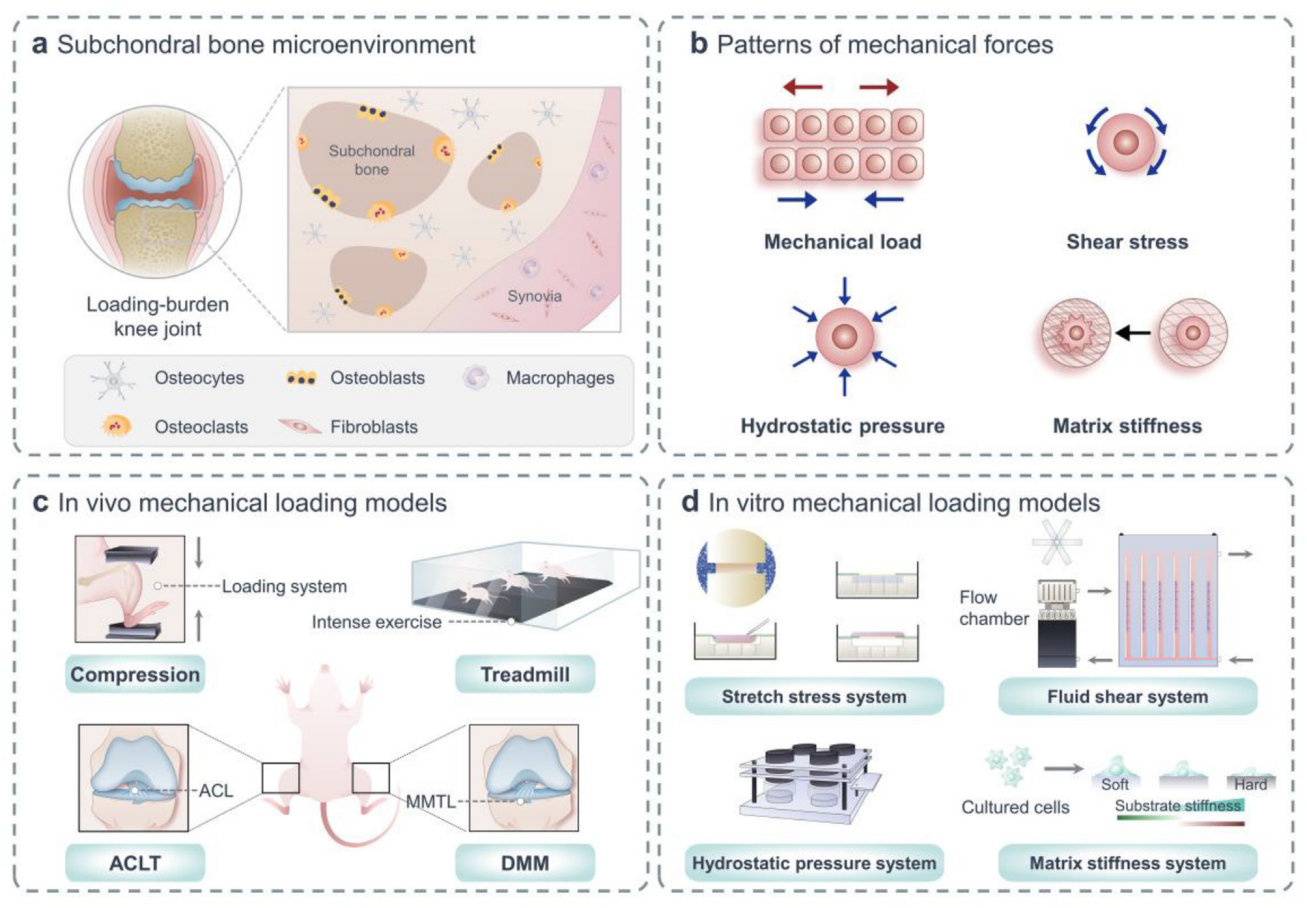
Structural basis of mechanics in skeletal cells and models commonly used in mechanobiology. (a) Subchondral bone microenvironment contains osteocytes, osteoblasts, osteoclasts *et al.*, all of which are sensitive to mechanical stimulation. (b) Mechanical stimuli applied to cells include mechanical load, shear stress, hydrostatic pressure, and matrix stiffness. (c) *In vivo* experiments: cycling mechanical compression is performed on the limb using a computational control machine; OA model is established by simulating the process of joint damage in running experiment; microsurgical techniques are used to perform ACLT and DMM models under direct visualization. (d) *In vitro* experiments: multi-channel cell stretch stress loading device can load uniaxial and biaxial stretches on cell and tissue cultures; multi-flow field cell fluid shear force loading culture and real-time observation/analysis system provide cells with various forms of fluid shear forces; continuous flow constant pressure for cell culture system uses hydrostatic water flowing into petri dishes to maintain target temperature, humidity, and carbon dioxide levels; isolated cells are seeded onto a polyacrylamide gel that mimicked different matrix stiffness.

The joint is a complex load-bearing structure of the human body that senses various mechanical forces (mechanical stress, fluid shear, tensile, *etc*.). It has been established that certain tissues such as subchondral bone and articular cartilage exhibit differences in their sensing tissues, mechanical force patterns, and cellular mechanosensing capabilities.^1^ Although cartilage degradation is a crucial characteristic of OA, it is now considered that the whole joint, especially the subchondral bone, is involved in the mechanical pathogenesis of OA. The microstructural changes of subchondral bone in joints suffering from OA include early bone loss, late bone sclerosis, and histopathological modifications (formed by subchondral bone cysts, bone marrow edema-like lesions), as well as osteophytes. These pathological processes involve the coupling and uncoupling of mechanically sensitive bone cells (such as osteocytes, osteoblasts, and osteoclasts) in subchondral bone microenvironment (Fig. 1a).^2^

In the latest years, great emphasis has been positioned on the characterization of mechanical microenvironment at cellular levels, forces recognized by mechanosensitive cells, and integrated cellular responses. Meanwhile, there is a developing consciousness of the significance of mechanical microenvironment in regulating and figuring out the orchestration of bone buildings. Bone tissue responds to mechanical stimuli of different types, including mechanical load, shear stress, hydrostatic pressure, matrix stiffness.^3^ Osteocytes are key cells that coordinate bone mass and structure by the biomechanical regulation to achieve efficient weight-bearing.^4^ Mechanical loading of osteocytes will increase intracellular Ca^2+^ concentration, and Ca^2+^ oscillations in bone cells set off the launch of downstream signaling molecules. Subsequently, secreted molecules regulate peripheral osteocyte function and affect osteogenesis and osteoclast activity to modulate bone homeostasis.^5^ Skeletal homeostasis is mediated through the use of the balance between osteoclast-mediated degradation of neighborhood bone matrix and osteoblast-mediated formation of new bone matrix. At the mechanical level, transient receptor potential channel Piezo1 in osteoblasts regulates the transcriptional co-activators Yes-associated protein (YAP)-dependent expression of type II and IX collagen in response to mechanical load. In turn, these collagen subtypes regulate osteoclast differentiation, which confirms Piezo1 as a mechanical bone sensor for regulating bone homeostasis.^6^ Intriguingly, numerous studies have shown that evaluating the influence of various mechanical forces on osteoclasts is essential for further study of OA pathogenesis.^7^

In brief, mechanical pressure has a significant impact on the physiological and pathological processes of joints, and definitely performs a position in the progression of OA. In this review, we introduce mechanical risks as the cause in the process of OA, and discuss mechanobiology researches of loading devices *in vitro* and *in vivo*. In mechanisms, there exist a variety of mechanoresponsive signaling pathways in mechanosensitive bone cells in subchondral bone microenvironment, which are related to the development of OA. A full grasp of the molecular groundwork of how bone responds to mechanical stimuli will assist to discover new procedures to the remedy of OA. Cumulative attention has been paid to the use of mechanical cues to improve bone health through exercise, surgery or targeting mechanical homeostasis in subchondral bone microenvironment as a therapeutic measure.

## 2 Mechanical forces on the joint

The mechanical environment of the joint is a dynamic surroundings of biophysical stimuli, including strain, stress, shear, pressure, fluid flow, flow potential and acceleration, and different types of cells are subject to different forms of perception (Fig. 1b).^8^ OA occurs at the abnormal biomechanical situations inside the joint, such as overloading, anterior cruciate ligament (ACL) and meniscus injuries, and ageing, particularly at high mechanical loading regions. Such abnormal biomechanical conditions are associated with subchondral bone disorder. As an interactive tissue unit, the bone-cartilage complex, which fuses articular cartilage, calcified cartilage and subchondral bone, is the focal site for the development of OA.^9^ Excessive mechanical stimulation will change the cell state, resulting in a decrease in the elastic modulus of cartilage and an increase in the elastic modulus of subchondral bone, which will interact with each other and eventually lead to OA.^10^ The stiffness of the subchondral bone increases, making it unable to absorb and buffer the load when the articular cartilage is loaded, leading to the deterioration of OA.^11^ Excessive mechanical load leads to an inflammatory response in the cartilage that activates mechanosensory receptors and signaling pathways, resulting in the production of catabolic enzymes that degrade functional cartilage extracellular matrix.^12^ The important mediators of mechanical inflammation include transforming growth factor-β (TGF-β), p38 mitogen-activated protein kinase (MAPK), c-Jun N-terminal kinase (JNK), YAP/transcriptional coactivator with PDZ-binding motif (TAZ) and nuclear factor-kappaB (NF-κB).^13^ These signals eventually lead to the over expression of inflammatory soluble mediators such as prostaglandins (PGEs), chemokines and cytokines when a certain threshold is reached, which eventually lead to cartilage degradation.^14^

Scholars have used various methods to simulate the effect of mechanical forces on the joint to explore the corresponding mechanical signal transduction pathways. Over the years, nonsurgical, mechanical pressure-based models of joint injury have been increasingly used for studying the pathogenesis and remedy of OA. Common non-invasive models are intra-articular tibial plateau fracture, cyclic articular cartilage tibial compression, and ACL rupture via tibial compression overload. Periodic articular cartilage tibial compression is used to study long-term effects of injury (increased bone remodeling and osteophytes) by applying cycles over a period of time and by adjusting load on the joint.^15^ What's more, a single cycle with a load of 12 ​N and a velocity of 500 ​mm/s was applied to the tibia to induce immediate severe injury and subsequent OA phenotypes.^16^ Models utilizing mechanical testing systems are highly reproducible, require little expertise, and lead to predictable episodes of osteoarthritic pathology on a rapidly progressive schedule.^17^^,^^18^ In contrast, surgically-induced OA models such as anterior cruciate ligament transection (ACLT) and destabilization of the medial meniscus (DMM) have been used to find out the pathogenesis of OA, such as subchondral bone changes.^19^ Through an experimental study in which male Wistar rats underwent DMM surgery on their right knee, Iijima *et al.* examined the dose–response relationship of exercise loading in the cartilage-subchondral bone unit and subjected to 12 ​m/min moderate or 21 ​m/min intense treadmill exercises for 30 ​min/day, 5 days/week for 4 weeks (Table 1).^20^

**Table 1:** Experimental conditions for *in vivo* mechanical loading models.

Animal model	Equipment	Body part	Mechanical force	Period	Brief summary	Refs.
Lewis rats	Non-Invasive Knee Injury device	Tibia	5.4 ​N	15–25 ​s followed by a single 3.26 ​± ​0.28 ​N/s ramp in compressive load	Non-invasive knee injury models produce consistent ACL breakage and lead to conventional OA pathology without directly damaging other tissues.	^17^
Mice	Loading system used for cyclic compression	Lower leg	7–10 ​N	1200 cycles per day, 5 days per week for multiple weeks	Joint injury can be induced by cyclic compression load or acute overload to induce ACL rupture.	^18^
Wistar rats	DMM surgery	Knee	21 ​m/min intense treadmill exercise	30 ​min/day, 5 days/week for 4 weeks	Exercise modulates the development of post-traumatic OA in a dose-dependent manner during DMM surgery.	^20^

Both invasive and non-invasive models have advantages and disadvantages. Comparatively speaking, invasive experiments are easy to perform and can induce OA quickly, ensuring that studies can be conducted in a shorter time frame, and surgically induced models have been used to study the pathogenesis of post-traumatic OA, such as subchondral bone changes.^16^ However, a weakness of induction models is that they do not correlate with natural degenerative changes in human degenerative OA. Noninvasive animal models may additionally be a suitable preference for studying OA due to their magnificent potential for enhancing open access studies and are dependable tools for studying early modifications in OA that are not viable in invasive (induction models) studies.^21^ Noninvasive models have a low risk of infection and are often used to study early changes and the effects of early treatment interventions. Besides, results from noninvasive animal models are highly reproducible.^21^ However, noninvasive models still have limitations. The equipment of non-invasive models is not universally available and depend on the proficiency of the technician. In addition, the accuracy of the instrument and the angle of knee flexion may affect the experimental results, resulting in different results observed between similar studies. It has been documented that age, sex (hormonal status), and mouse strain can also have an effect on the effects of noninvasive model.^22^^,^^23^

## 3 Perception of subchondral bone cells to mechanical force patterns

Subchondral bone always maintains a dynamic balance in tissue remodeling, and mechanical load performs a key function in bone remodeling. Within a certain range, the mechanical sensitivity of subchondral bone is enhanced; beyond limits, the cells begin to compensate but still maintain normal function. Once decompensated, the signaling pathway changes and pathological features appear.^24^ During the pathological progress of OA, in addition to the diversity of forces, as the subchondral bone exhibits different degrees of damage, the ability of each cell to sense mechanical force is altered. Current evidence suggests that various cell types work synergistically to yield adaptive changes in response to alterations in the mechanical environment by coordinating several mechanical parameters in the joint, both temporally and spatially.^25^ Thus, several biomechanical ‘joint motion’ devices to study the effect of joint stress on cells *in vitro* are urgent for development (Table 2).

**Table 2:** Experimental conditions for *in vitro* mechanical loading models.

Cell type	Mechanical stimulation	Duration	Response	Refs.
MC3T3-E1 osteoblast	Fluid shear stress	0.70 ​Pa, 0.70 ​± ​0.31 ​Pa, 0.70 ​± ​0.70 ​Pa, 5 or 9 ​Hz	NO production is a parameter of osteoblast activation linearly dependent on the rate of fluid shear stress.	^27^
Osteocyte	Dynamic substrate strain; fluid flow	0.1–10% 1 ​Hz; 2 ​N/m^2^ 1 ​Hz	Fluid flow plays a greater role than matrix deformation in bone cell mechanical transduction associated with bone adaptation to conventional load.	^28^
Osteocyte	Fluid pressure	8 ​dyn/cm^2^	During cyclic loading, tracer transmission increases with increasing load amplitude and permeability, and decreases with increasing loading frequency.	^31^
Normal human osteoblast	Shear stress	20 ​dyn/cm^2^	Fluid flow shear stress induces the proliferation and differentiation of osteoblasts through various signaling pathways.	^34^
MG63 osteoblast-like cell	Shear stress	0, 1, 5, 14, and 30 ​dyn/cm^2^	Decrease in shear force-mediated osteoblast differentiation may be due to the increased production of PGE2.	^35^
Osteoclast precursor RAW264.7 cell	Low-fluid shear stress	0.1–0.7 ​Pa	Osteoclast precursors sense fluid shear stress gradients and tend to actively migrate to regions with low fluid shear stress, which are regulated by Ca^2+^ signaling pathways.	^42^
Co-cultured osteoclast	Mechanical stress	Cyclic loading at 1 ​Hz	Mechanical stress-induced TGF-β1 overexpression of osteoclast is the cause of OA chondrocyte apoptosis and cartilage degeneration.	^46^
Bone marrow-derived macrophage and RAW264.7 monocyte	Different stiffness degrees	Substrates 1:5 (E ​= ​∼4.05 ​MPa) and 1:45 (E ​= ​∼0.1 ​MPa)	Harder PDMS substrates accelerate osteoclast differentiation.	^114^
Osteoclast of rabbits	Intermittent tensile strain	Magnitude 1730 μϵ	Mature osteoclasts independently sense mechanical stimulation and upregulate bone resorption activity.	^115^

### 3.1 Osteocytes

Osteocytes, the most numerous cells in the bone, with chemosensory and endocrine functions embedded between mineralized tissues of bone and are regarded as the primary facilitators of load-induced bone remodeling. Osteocyte syncytium senses biomechanical forces on bone through tubule networks and intercellular interstitial junctions, mainly affected by stress and fluid shear forces.^26^ Fluid flowing through the pericellular matrix causes 1-2 order of magnitude large than the typical tissue stress and adequate to provoke intracellular signaling.^26^ When bones are subjected to mechanical stress, fluid flows through tiny channels in the bone matrix, causing shear stress in the cell membranes that activate osteocytes. Klein-Nulend *et al.* demonstrated that in response to mechanical stimulation activated osteocytes produce signaling molecules such as NO, which regulate the activity of bone forming osteoblasts and bone resorption osteoclasts, thus coordinating bone adaptation to mechanical load.^27^ You *et al.* found that both cyclic strain and fluid flow could induce Ca^2+^ transients in osteocytes, but the fluid flow was 5 times more likely to induce Ca^2+^ transients than a substrate strain at presumed physiologic levels by a parallel plate flow chamber and computer-controlled stretch device. Fluid flow also affected osteopontin (OPN) expression more than substrate strains.^28^ Moreover, osteocytes reply immediately to mechanical stimuli and produce signaling molecules that modulate the activity of osteoblasts and osteoclasts, thus converting mechanical stimuli to cellular signals. Osteocytes promote osteoclasts activity in front of the dissected cone with low stress and stimulate osteoblastic bone formation round the closed cone with high stress.^29^ Although typical *in vitro* studies on osteocytes, such as the MLO-Y4 cell line, have been conducted in two-dimensional (2D) environments rather than within the three-dimensional (3D) context of bone matrix, the mechanical stimulation and signaling molecules conducted in these experiments have provided valuable insights into bone cell morphology and flow in different environments.^30^^,^^31^

### 3.2 Osteoblast lineage cells

Osteoblasts can be mechanically stimulated *in vitro*, mainly by fluid flow stress, basal stretching and four-point bending. The response of osteoblasts depends on the duration, type and level of stress.^32^ Emerging research have substantiated that mechanical stimulation have an effect on the differentiation and metabolism of osteoblasts.^33^ Intriguingly, it has been demonstrated that osteoblast differentiation is induced by mechanical stimulation, triggering oxygen sensing mechanisms, paracrine vascular endothelial growth factor (VEGF) and placental growth factor (PIGF) signaling, thereby affecting the differentiation and proliferation potential of osteoblasts.^34^ Many studies have reported the proliferation or DNA content of primary osteoblasts increased significantly under constant fluid flow by flow loop apparatus or surface roughness.^33^^,^^35^ Mechanical load also affects the role of osteoblasts in bone formation and maintenance of bone homeostasis. When the bone is subjected to an external load, fluid flow around osteoblasts creates fluid shear stress, stimulating osteoblasts to increase metabolism. PGE2 release is a significant load-induced response in osteoblast-like cells. Insulin-like growth factor-I (IGF-I) and IGF-II promote Osterix expression in osteoblasts, and induce osteoblasts *in vitro* and temporarily increase bone mass *in vivo.*^36^ Specifically, 15 ​min of gravitational pressure pulses applied to osteoblasts by using cellular g-load apparatus increased osteoblast proliferation after 24 ​h, similar to *in vivo* physiological levels seen during walking or running.^37^ However, excessive stress on osteoblasts induces the expression of bone morphogenetic protein (BMP) extracellular antagonists, thus inhibiting the formation of osteoblasts.^38^ Moreover, continuous compression *in vitro* can induce osteoblasts to produce inflammatory cytokines and their receptors, which may be associated to the signaling pathway of RANKL and its receptor RANK, which system is responsible for inducing osteoclastogenesis.^39^

### 3.3 Osteoclast lineage cells

Osteoclasts are one of the components of bone tissue composed of multinucleated giant cells and are the only cells in the human body that can decompose and absorb bone. However, the mechanics underlying bone resorption by osteoclasts have been less studied. Over-inhibition of osteoclasts in mechanical studies may be due to their complex differentiation and flexible structure, which increases the difficulty in targeting the mechanical structure. Importantly, the effects of mechanical tension on osteoclast activity vary depending on the intensity and duration of the stimulus.^40^ Moreover, the fluid shear force can induce osteoclast proliferation, formation and differentiation. Proper mechanical stimulation of bone formation also provide RANKL and osteoprotegerin (OPG) for osteoclast maturation. Mechanical stress on osteoclasts can reduce their activity and inhibit bone resorption.^41^ Mechanical control of osteoclast function appears to be mainly achieved by regulating osteoclast recruitment through the expression of bone progenitor cell lineage of RANKL. In turn, cells of bone progenitor cell lineage are located in a mechanically active environment and respond to mechanical cues through proliferation, differentiation, and changes in differentiation function.^8^ Recent researches have proven that osteoclast precursors under gradient fluid shear stress have a tendency to actively migrate to low-fluid shear stress regions regulated by means of Ca^2+^ signaling.^42^ Mechanical stress induction can regulate tartrate-resistant acid phosphatase (TRAP), OPG, RANKL using a novel compression model embedded osteoclast in 3D gels and control osteoclast-related factors (such as cathepsin K (CTSK), macrophage colony-stimulating factor (M-CSF) and matrix metalloproteinase-9 (MMP-9)) by low-magnitude high-frequency vibration.^43^^,^^44^ Moreover, TGF-β1 is regarded as a central regulator of subchondral bone remodeling under mechanical stress, which is also a key indirect mediator of OA.^45^ Additionally, mechanical stress triggers the upregulation of osteoclastic TGF-β1, which contributes to articular cartilage degeneration and OA-like changes, including synovial hyperplasia and knee osteophyte formation in C57BL/6 mice.^46^ In co-cultured osteoclast precursors and OA synovial fibroblasts, the production of OPG in TGF-β-induced synovial fibroblasts inhibited osteoclast formation. In addition, blocking TGF-β activity in OA joints promotes osteoclast generation and inhibits osteophyte formation.^47^ Sometimes the results may be different or even opposite, which may be attributed to the heterogeneity in stress loading methods and stress parameters or osteoclasts at different stage of OA (Fig. 1c–d).

## 4 Mechano-signaling in subchondral bone cells

The research of biomechanics has been extended from organ/tissue level to cellular/molecular level. Mechanobiology studies include how cells perceive mechanical signals, and how mechanical signals stimulate protein expression and cell differentiation.^48^ Articular cartilage is suitable for low-friction motion and weight-bearing. Under mechanical stress, growth factors sequestered in the extracellular matrix activates a variety of intracellular signaling pathways, including protein kinase C (PKC), MAPK and phosphoinositide 3-kinase (PI3K)-protein kinase B (Akt) pathway. Integrin activation triggers biochemical signal transduction and direct mechanical cell deformation through cytoskeleton contraction. Physiological load induces anabolic responses in chondrocytes through the activity of TRPV4 ion channels, but Piezo channels is involved in the mechanical transduction of harmful mechanical stimuli, specifically Piezo1 and Piezo2.^49^ The main biological functions of subchondral bone are absorbing stress, buffering shock and maintaining joint morphology. The effects of mechanical factors on joint health, adaptation, and damage (decompensation) mechanisms are complex and multifaceted, and result from the comprehensive effect of various mechanical signaling pathways in the microenvironment.^50^ Therefore, paying attention to mechano-signaling in subchondral bone cells can help explain the microstructure and histopathological changes of subchondral bone at different stages of OA progression and may be used as a therapeutic target to block OA progression (Table 3).

**Table 3:** Mechanosensing structures and mechanotransduction signaling pathways of the subchondral bone microenvironment.

Cell type	Mechanical stimulation	Mechanical signaling	Major outcomes	Refs.
Osteocyte	Fluid flow stress	Integrin αvβ3	Focal mechanical stimulation of osteocytes occurs along processes but not cell body and αvβ3 integrin plays an essential role in osteocyte activation.	^55^
	Fluid-flow induced forces	Matrix-dependent adhesion	Direct interstitial junctions between MLO-Y2 cells in a connected osteocyte network are coupled with extracellular purinergic P4 receptor signaling in response to mechanical signals.	^56^
	ECM stiffness	YAP/TAZ	YAP/TAZ activity is induced by ECM hardness and cell diffusion in stressed fiber and cytoskeletal tension.	^58^
	Dynamic fluid flow	Primary cilia	Primary cilia in bone transfer fluid to cellular responses in osteocytes independent of Ca^2+^ fluxes and stretch activated ion channels.	^61^
	Static and fluid flow	Piezo1	Over-expression of Piezo1 in MLO-Y4 cells increases the expression of Ptgs2 and Tnfrsf11 ​b and enhances the response of the fluid shear stress.	^90^
Mesenchymal stem cell	Hydrostatic pressure	Piezo1	0.01 ​MPa hydrostatic pressure is the most suitable to induce MSCs differentiation into osteoblast lineage cells and induce osteoblast differentiation.	^88^
Osteoblast	Shear stress	TRPM7	TRPM7 is mechanically sensitive to shear forces of 1.2 ​Pa, much lower than the recently reported pressure loads of 98 ​Pa, and mediates different mechanical transduction pathways.	^92^
	Hypo-osmotic stress	TRP channel	Hypo-osmotic stress induces Ca^2+^ influx through TRPM3 and TRPV4 to regulate RANKL and NFATc1 expression.	^96^
	Tensile stress	Wnt/β-catenin	Tensile stress can promote the expression of β-catenin protein in preosteoblasts at the initial stage of the experiment, but reduce at 12 ​h and 40 ​h after the end of periodic loading.	^100^
	Strain/stretching	Runx2	Runx2 affects osteoblast function and mediates mechanical transduction by binding to osteoblast-specific cis-acting element 2.	^108^
Osteoclast	Substrate stiffness	Cytoskeletal arrangement	Extracellular substrate hardness is an essential determinant of osteoclast differentiation and function, and stiffer stiffness accelerates osteoclast differentiation.	^114^
	Mechanical stretching	SA-cat channel	Mature osteoclasts respond to mechanical stretching through mechanisms involving the SA-cat channel and thus up-regulate bone resorption activity.	^115^
	Fluid flow	STIM1 and TRPV4	Intracellular Ca^2+^ oscillations in osteoclasts mechanically stimulated by fluid shear force induce STIM1 to be highly expressed in early osteoclasts and TRPV4 to be highly expressed in late osteoclasts.	^116^
	Substrate rigidity	Podosomes	By detecting the stiffness and topography of the matrix, the podosomes mechanically sense both intracellular and extracellular signals generated downstream.	^119^

### 4.1 Osteocytes’ perception and response to mechanical signals

#### 4.1.1 Cytoskeleton and cilia

It is the cytoskeleton, especially its mechanical properties, that determines the origin of cellular mechanical properties and how they respond to extracellular mechanical stimuli. For osteocytes, three sorts of cytoskeletal filaments make up the bulk of the cell: microtubules (MTs), actin filaments (F-actin), and intermediate filaments (IFs).^51^ Normative osteoblast morphology and mechanical responses are maintained by these three cytoskeletal filaments. Integrins are heterodimeric transmembrane receptors composed of α and β subunits. These molecules functionally link extracellular matrix (ECM) to cytoskeleton, thereby transforming mechanical stimulation from the extracellular environment to intracellular components.^52^ Integrin αvβ3 promotes Ca^2+^ signaling pathway diffusion, activates PI3K/Akt signaling pathway, and increases the production of anabolic factors. Fluid shear stress stimulate Connexin43 hemichannels (Cx43 ​HCs) to open and activate osteocyte αv and α5 integrins to release anabolic factors.^53^ Cytoskeletal networks are characterized by the mechanical properties of the cytoskeletal components at one end or side. It has been established that three cytoskeletal components have different mechanical responses to osteocytes.^54^ IFs have been mainly reported in cell bodies, while F-actin and MTs have been primarily documented in cell bodies and dendrites. Current evidence suggests that osteocytes primarily use focal adhesion on dendrites to receive mechanical signals,^55^ transduce these signals via F-actin and MT cytoskeletons or Ca^2+^ signals, and initiate nuclear reactions to regulate the target genes expression and the response of certain subcellular organelles activated by secondary messengers (*e.g*., Ca^2+^ and ATP).^56^ What’ more, changes in intercellular contact, cell stretching, cell tension, ECM hardness, and cell geometry modulate Rho GTPase activity, leading to actin cytoskeletal remodeling. The actin cytoskeleton controls the nuclear-cytoplasmic shuttle and transcriptional activity of YAP/TAZ through LATS1/2-dependent and LATS1/2-independent mechanisms.^57^^,^^58^

Some specialized structures of the osteocyte skeleton, such as primary cilia produced by F-actin-related MTs and focal adhesions (FAs), have been broadly identified as main mechanical sensors in osteocytes.^59^ A typical cilium structure is composed of an MT-based central axial filament that emerges from the central basal body of the centromere-derived MT tissue and extends from the specialized plasma membrane into the extracellular area, consisting of nine MTs in a “9 ​+ ​0” shape.^60^ The primary cilia of osteocytes are closely related to bone development and mechanical conduction. Primary cilia are reportedly involved in the mechanical conduction of osteocytes *in vitro* and *in vivo*. Under real-time imaging, the primary cilia deflected in the steady flow direction of 0.03 ​Pa and retracted after stopping. In MC3T3-E1 and MLOY4 cell lines, damage to primary cilium structure by chloral hydrate treatment or Polaris siRNA reduced cell response to flow, including mechanically-induced reductions in OPN mRNA expression, extracellular PGE2 levels and OPG/RANKL mRNA ratio, further affecting the transmission of mechanical signals.^61^ Cell culture experiments have shown that primary cilia in osteocytes can sense fluid flow through the lacuna-tubule network, and this stimulation is translated into osteogenic responses in osteocytes via biochemical signals.^62^ Primary cilia knockout in osteocytes has been proven to decrease bone formation in response to load *in vivo.*^63^ Although the exact mechanism underlying primary cilia-mediated mechanical induction in osteocytes remains unknown, many studies have confirmed the relevant signaling molecules. Some studies have identified molecular mechanisms involving cyclic adenosine monophosphate (cAMP) and adenylate cyclase 6 (AC6) that link primary cilia and osteocyte mechanical transduction, but the specific mechanisms remain unclear. Primary cilia mediate osteogenic and anti-absorption responses to osteocytes flow, such as PGE2 release, OPN and cyclooxygenase 2 (COX-2) expression, and increased OPG/RANKL mRNA ratios.^60^

#### 4.1.2 Ion channels

The earliest sign of mechanoconduction is an increase in intracellular Ca^2+^ concentration within 1 ​min following mechanical stimulation. Piezo1 is a curved channel in high contact with cell membranes. Osteocyte membrane tension is increased by mechanical stimulation, which further triggers Piezo1 activation.^64^ Ca^2+^ fluxes induced by mechanical stimulation come from external fluids and media and internal Ca^2+^ storage sites such as the endoplasmic reticulum. Ca^2+^ mobilization activates downstream effector factors, including actomyosin, ERK1/2, FAK, PGE2 and OPN and regulates the release of ATP from osteocytes during mechanical stimulation.^65^ Moreover, among the various regarded mechanosensitive channels, the significance of transient receptor potential (TRP) channels is extensively studied. Osteocytes respond to fluid shear stress by microtubule network dependent activation of reactive oxygen species (ROS) produced by NADPH oxidase 2 (NOX2) and subsequent opening of TRPV4 cation channels, resulting in Ca^2+^ inflow. It can further induce changes in the expression of Osterix, OPG, and sclerostin (SOST) genes which are important for maintaining bone homeostasis.^66^

#### 4.1.3 Connexin 43

Osteocytes express a number of types connexins, including Cx40, Cx43, Cx45, Cx46, and Cx37. Cx43 is a highly expressed gap junctions (GJ) protein in bone,^67^ and Cx43-dependent gap junctional intercellular communication (GJIC) and semi-channel help coordinate bone remodeling in response to anabolic elements and mechanical load.^68^ Cx43 deficiency plays an important role in bone development and homeostasis and desensitizes the bone to mechanical load and unloading effects.^69^ Cx43 is a transmembrane linker protein, six Cx43 surround to form a transmembrane linker, namely half channel. Two linkers on adjacent cell membranes butt to form a GJ, and a pore with a diameter of about 1.5 ​nm is formed in the center, allowing the intercellular exchange of molecules with molecular weight less than 1.2 ​kDa.^70^ This channel not only enables communication connections between cells, but also releases intracellular small molecules into the extracellular, helping to coordinate bone remodeling in response to anabolic factors and mechanical load.^71^ The expression and distribution of Cx43 in osteocytes change when subject to mechanical stimulation. After sensing fluid flow stress, Cx43 move toward the synaptic direction of cell membrane in osteocytes, rapidly increasing intercellular coupling.^72^ In addition, mechanical stimulation also induced Cx43 hemichannel opening. When osteoid cells MLO-Y4 receives fluid shear force, it will induce the opening of semi-channel and release bone remodeling factors such as PGE2, ATP, NO.^73^ The released PGE2 further enhances the expression of Cx43 in osteocytes, forming a positive feedback loop that ultimately leads to more extensive and more intense responses. Moreover, oscillating fluid flow promotes the formation of new GJs without affecting dye transfer between established GJs, which depends on the ERK1/2-MAPK pathway.^74^

#### 4.1.4 Wnt pathway

There is a rich literature available substantiating that the Wnt pathway performs an essential function in osteocytes’ differentiation, proliferation and apoptosis.^75^ The rules of the Wnt/β-catenin signaling pathway is ordinarily decided via proteins appearing as Wnt aggressive binding (especially sFRP protein family) or low-density lipoprotein receptor-related protein 5 (Lrp5) level, such as osteoblast-specific proteins, sclerotin (the SOST gene product), Wise and Dikkops (DKK) proteins, in particular DKK-1 and DKK-2.^76^ DKK-1 is mainly expressed by osteocytes, which is a negative regulator of Wnt signaling. Mechanical loading inhibited the expression of DKK-1 and promoted the upregulation of the Wnt signal in osteocytes. Sclerosing proteins are reportedly produced by mature osteocytes, inhibiting Wnt/β-catenin signaling by binding Lrp5 and blocking Wnt binding.^77^ Mechanical forces decrease SOST expression and increase periostin (Postn) expression, while the contrary is unloading.^78^ Interestingly, the downregulation of SOST in osteocytes is a mandatory step in the mechano-transduction cascade to activate Wnt signaling and guide osteogenesis. In addition, SOST acts directly on osteocytes to attenuate the effect of mechanical load on bone.^79^

**Fig. 2:**
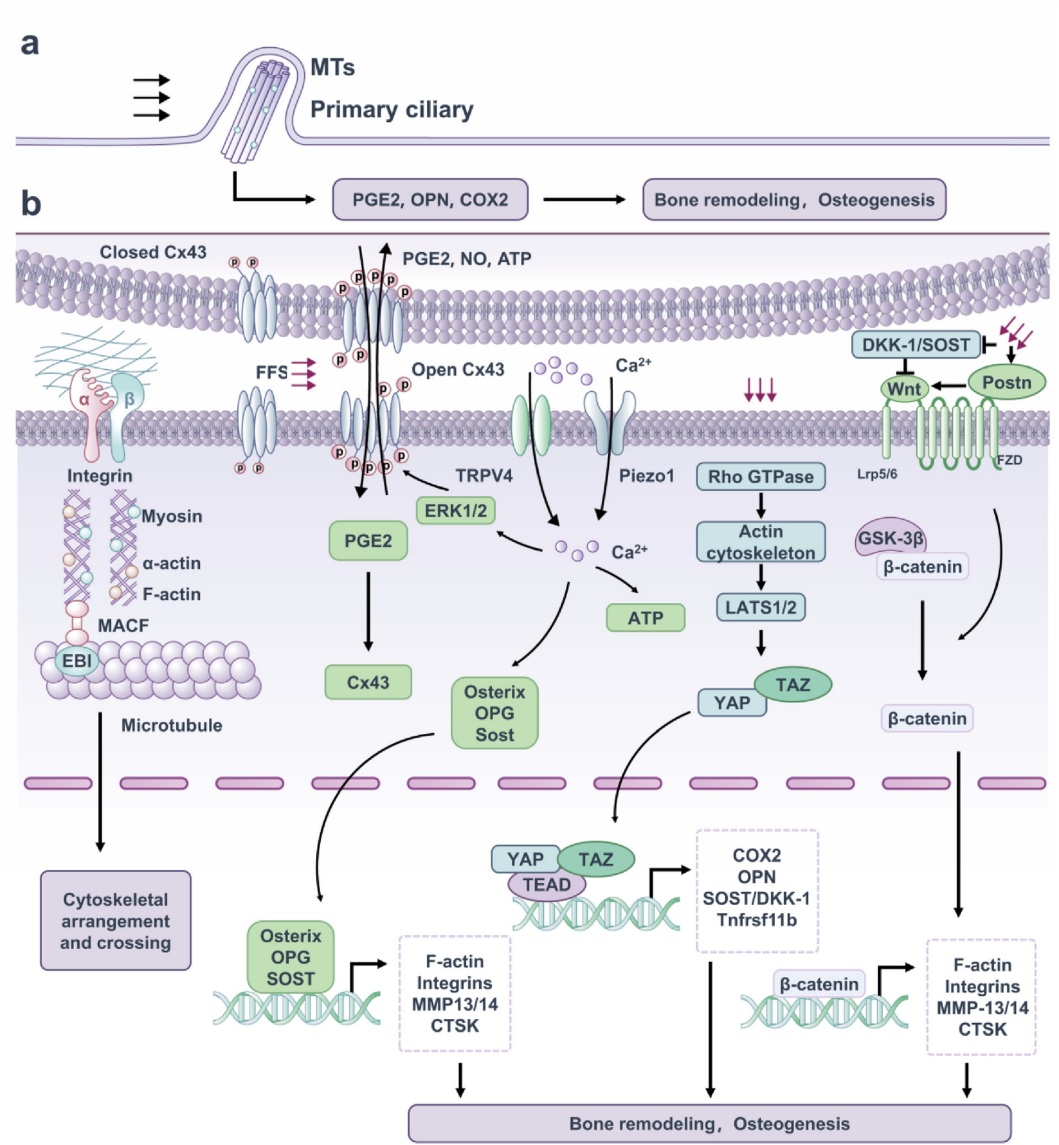
Mechanosensitive structure and signaling pathway of osteocytes. (a) Primary cilium is a special cell protrusion structure consisting of 9 doublet MTs in the shape of a “9 ​+ ​0” pattern, which transmit mechanical signals via the participation of ciliate protein. (b) Integrin adhesion complexes connect ECM and F-actin cytoskeleton and enhance activation of downstream pathways. Upon subjected to mechanical stimulation, Cx43 protein is phosphorylated, the linker is turned on, causing the increase of PGE2 and maintaining bone remodeling. The earliest event in osteocyte mechanical transduction is the increase in intracellular Ca^2+^ concentration. This process is mobilized by which mechanical stimulation increases the osteocyte membrane tension, further inducing the opening of Piezo1, TRPV4 channels, Ca^2+^ influx, and further promoting osteogenesis and maintaining bone homeostasis. Mechanical stimulations modulate Rho GTPase activity, leading to actin cytoskeletal remodeling, which controls the nuclear-cytoplasmic shuttle and transcriptional activity of YAP/TAZ. Typical Wnt/β-catenin pathway is activated by Wnt ligand binding to a co–receptor complex consisting of Lrp5 or Lrp6 and FZD, and GSK-3β is phosphorylated to release the captured β-catenin. As a result, free β-catenin is transferred to the nucleus to induce downstream gene transcription, which promotes bone formation.

Importantly, the Wnt signaling pathway plays various roles in skeletal homeostasis. The typical Wnt signaling pathway begins with the binding of the Wnt ligand to the Frizzled (FZD) and Lrp5/6 receptors located on the cell membrane. Activation of the Wnt signal promotes the accumulation of β-catenin by inhibiting the GSK-3β-induced phosphorylation of β-catenin, after which dephosphorylated β-catenin is translocated into the nucleus and further induces the transcription of lymphoid enhancer factor/T-cell factor (LEF/TCF) response gene.^80^ As the main intracellular signal transducers of Wnt signaling pathway, β-catenin is one of the cores of mechanical conduction.^81^ It has been reported that Wnt activity increases during mechanical loading and decreases during unloading.^82^ Mechanical strain can induce mesenchymal stem cells (MSCs) to differentiate from lipids to osteoblasts by preserving β-catenin in the nucleus.^83^ In this respect, mice lacking β-catenin in osteocytes confirmed severe osteopenia and vulnerable bones and died prematurely.^84^ Numerous studies support the hypothesis that the Wnt/β-catenin signaling pathway is a physiological response to loading, and activation of the Wnt/β-catenin pathway improves the mechanical sensitivity of osteoblasts/osteocytes. OA is associated with irregular osteocytes activity and altered protein expression at the molecular stage.^84^ The Wnt/β-catenin pathway responsible for osteogenesis and bone remodeling is significantly upregulated in OA subchondral bone. SOST as the major Wnt inhibitor expresses in calcified cartilage and subchondral bone, and its deficiency has been associated with OA, possibly through activation of Wnt signal transduction.^85^ Increases in subchondral bone quantity fraction, trabecular bone thickness, and strange bone mineral density distribution have been discovered in each OA and SOST defect fashions.^86^ This variation is considered load-dependent because higher bone mass is usually over-concentrated in load-bearing areas and relatively low in less or non-load-bearing areas (Fig. 2).^87^

### 4.2 Mechanotransduction in osteoblast lineage cells

#### 4.2.1 Mechanosensitive ion channels

MSCs are pluripotent stem cells that are precursors of osteoblasts. Many kinds of mechanical stimulation have been shown to influence the proliferation, development and differentiation of MSCs, including matrix mechanics (stiffness and topography) and external mechanical cues (hydrostatic pressure, fluid flow, compression, tension). Piezo1 channels in MSCs sense hydrostatic pressure and activate ERK1/2 and p38 MAPK signals, thus upregulating BMP-2. This upregulates Runt-related transcription factor 2 (Runx2) and Osterix, which contribute to the expression of osteogenic genes such as alkaline phosphatase (ALP) and type I collagen (COL1A1) and further promote osteoblast differentiation.^88^ Piezo1 and Piezo2 are fundamental to structure and preserve bones through mechanical forces; they convert fluid stress and ECM hardness into Ca^2+^ influx, activating the serine/threonine phosphatase calcineurin (CN),^89^ which dephosphorylates and promotes the transcription elements nuclear factor of activated T cells c1 (NFATc1), YAP, and β-catenin.^88^ Piezo1 is also a mechanically-sensitive ion channel in osteoblasts. It has been shown that defects in Piezo1 significantly reduced the bone mass and strength of mice and their ability to respond to mechanical stimuli. In addition, the loss of Piezo1 has been associated with increased osteoclasts. In contrast, feeding adult mice with Piezo1 agonist increased bone mass, simulating the effect of mechanical load.^90^ The osteoblast cell line regulates bone formation and metabolism by sensing and responding to changes in mechanical load. The deficiency of Piezo1 in osteoblasts leads to bone resorption increase, bone mass loss, and even spontaneous fracture.^91^ In addition, Piezo1 deficient mice are resistant to further bone loss and resorption caused by the offloading of their limbs, suggesting that Piezo1 can affect osteoblast-osteoclast crosstalk in response to mechanical forces. At a mechanistic level, Piezo1 in osteoblasts controls the YAP-dependent expression of type II and IX collagen in response to mechanical load. In turn, these collagen subtypes regulate osteoclast differentiation.^6^ Taken together, Piezo1 is regarded as the main bone mechanical sensor for regulating homeostasis.

TRP channels are broadly expressed cation channels that mediate various physiological stimulation, including canonical (TRPC), melastatin (TRPM), and vanilloid (TRPV) subtypes. Transient intermittent shear flow upregulates TRPM7 and induces translocation of TRPM7,^92^ phosphorylation of Smad1/5 and p38 MAPK, and upregulates Osterix, Distal-less homeobox 5 (DLX5), ALP and COL1A1. TRPM7 also delivers mechanical stimuli through its own actions, sensed by tension in lipid bilayers or poorly understood mechanisms through cytoskeletal tethers.^93^ This channel translates this stimulation into Ca^2+^ influx, triggering further Ca^2+^ release by inositol trisphosphate receptor 2 (IP3R2) in the endoplasmic reticulum via pulmonary lymphangitis carcinomatosis (PLC) and increasing nuclear localization of NFATc1, ultimately promoting osteogenesis.^94^ Members of the TRP superfamily, predominantly TRPVs, have been implicated in both Ca^2+^ homeostasis and bone metabolism. TRPV4 responds to a wide variety of stimuli, including mechanical stretching, endocannabinoids, hypotonicity, pH, pain, and cell swelling. Recently, it has been shown that activating mutations in the TRPV4 gene leads to several skeletal phenotypes in humans, including several dysplasias of the brachyolmia, spondylometaphyseal, Kozlowski and metatropic types. Both Piezo1 and TRPV4 are mechanical sensors dependent Ca^2+^ response in osteoblasts MC3T3-E1 that alter cell proliferation.^95^ TRPV4 is involved in the mechanosensory processes of osteoblasts and plays a vital role in bone remodeling. Studies in mouse osteoblasts have shown that hypo-osmotic stress induces Ca^2+^ influx through TRPM3 and TRPV4 to regulate RANKL and NFATc1 expression.^96^

#### 4.2.2 Wnt/β-catenin pathway

In osteoblasts, the Wnt/β-catenin signaling pathway is the most classical, and the cellular endocrine Wnt ligand binds to Lrp5/6 receptors via cell surface receptor FZD family to form a complex that causes the intracellular accumulation of β-catenin, thereby transmitting mechanical signals into the cells. Fluid shear force activates the Wnt/β-catenin signaling pathway and affects osteoblast differentiation.^97^ Moreover, the expression of β-catenin protein is enhanced by stress loading *in vivo* and *in vitro*, and the Wnt/β-catenin pathway increase the mechanical sensitivity of osteoblasts.^98^ Some studies have found that 15 ​min periodic tensile stress promotes the expression of β-catenin protein in preosteoblasts at the initial stage of the experiment, but the expression of β-catenin protein is significantly reduced at 12 ​h and 40 ​h after the end of periodic loading.^99^ β-catenin plays different roles in different stages of osteoblast development. The deficiency of β-catenin not only blocks osteoblast differentiation,^100^ but also leads to increased osteoclasts and severe osteopenia. Typical Wnt/β-catenin signaling prevents osteoblasts from differentiating into chondrocytes and promotes osteogenesis.^101^

#### 4.2.3 Other mechanical cascades

TGF-β/BMPs have widely recognized roles in bone formation during mammalian development and exhibit versatile regulatory functions in the body.^102^ The increased concentration of TGF-β in human OA subchondral bone induces the formation of Nestin+ MSC clusters, resulting in abnormal bone formation and increased angiogenesis.^103^ In ACLT rodent models, inhibition of TGF-β signaling plays a protective role by restoring coupled bone remodeling and reducing subchondral bone excessive angiogenesis.^104^ Experiments showed that cartilage degradation and excessive bone remodeling were associated with altered TGF-β signaling during the progression of OA in Dunkin-Hartley guinea pigs.^105^ As a multifunctional growth factor, BMP is essential for maintaining the balance of bone metabolism and performs an important position in bone development and fracture healing. BMPs bind to its receptor BMPR to promote osteoblast differentiation through protein signaling, among which Smad-Runx2 is one of the main pathways for BMPs to transmit signals to cells. Studies have shown that appropriate activity or pressure stimulation can promote fracture healing by enhancing osteogenic effect. 10% pressure (11.81 ​± ​0.42) kPa can promote the expression of BMP-2, Smad5, Runx2, and further promote the expression of ALP, COL1A1, osteocalcin (OC), OPN. The release of PGE2 is a significant load-induced response of osteoblast-like cells.^106^ Low-magnitude high-frequency vibration (LMHFV) stimulates osteoblast differentiation by activating the COX2-PGE2-EP (PGE2 receptor) pathway.^107^ PGE2 is produced by osteoblasts in response to physiological stress, growth factors, hormones, trauma, or inflammatory cytokines and induces osteoblasts to express cAMP-dependent IGF-I. IGF-I and IGF-II promote Osterix expression in osteoblasts, further triggering a transient bone mass increasing *in vivo.*^107^ In addition, NO, growth-related gene c-Fos, EGR-1 and autocrine bFGF are also upregulated, and MC3T3-E1 osteoblasts growth are induced. Specifically, 15-min pulses of gravity stress on osteoblasts, analogous to *in vivo* physiological levels discovered during walking or running, ought to extend osteoblast proliferation after 24 ​h. Therefore, appropriate mechanical forces can promote bone remodeling and increase bone density.^33^

Activation of intracellular signaling pathways and growth factors converge to activate the primary transcription regulator, transcription factors and Runx2 of osteoblast differentiation. Among the diverse stimuli that regulate Runx2 activity, mechanical load (strain/stretch) is one of the most critical signals linking Runx2 to osteoblast function and bone remodeling via mechanical transduction. Mechanical stretching triggers the Ras-ERK1/2 signaling pathway and enhances its transcriptional activity by phosphorylating Runx2, possibly through integrins αvβ3.^108^ It has been shown that mRNA and protein levels and the DNA-binding activity of Runx2 are enhanced by low-level mechanical stretching of cultured human osteoblasts. This forced firing of Runx2 induces aggregation via a specific MAPK pathway, as ERK1/2 MAPK is activated in a time-dependent manner and paralleled by an increase in Runx2 DNA binding potential. Although the loss of a Runx2 allele in heterozygous knockout mice doesn't affect bone homeostasis under physiological conditions, the loss of bone mass in Runx2 mice is exacerbated in the absence of mechanical stimulation.^109^

**Fig. 3:**
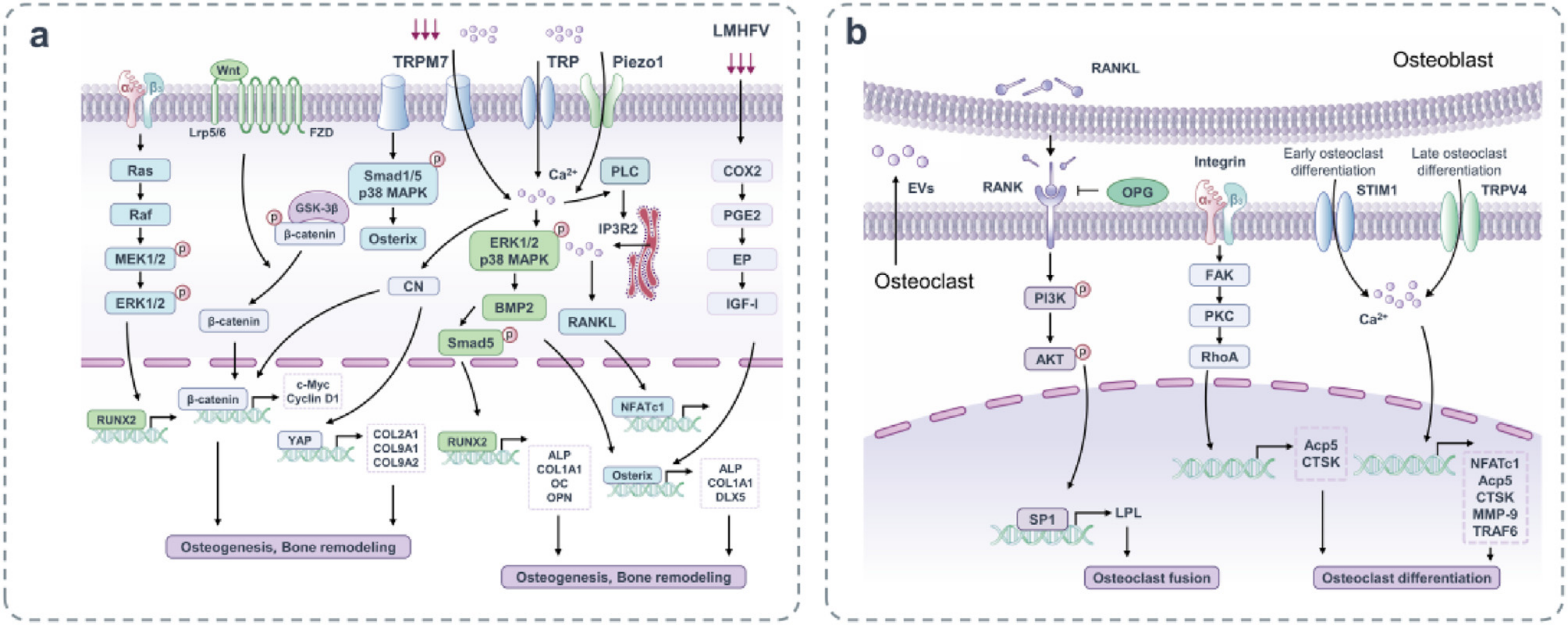
Mechanosensitive structures and signaling pathways of osteoblasts and osteoclasts. (a) Membrane binding receptors such as integrins, TRPM7, Piezo1 and so on are stimulated by mechanical forces to induce multiple transcription factors that regulate osteoblast differentiation and formation. Mechanical stimulation activates the typical Wnt/β-catenin signaling pathway and affects osteogenesis and bone remodeling. LMHFV activates the COX2-PGE2-EP pathway to stimulate osteoblast differentiation. (b) Integrin αvβ3 is activated by mechanical stimulation, followed by a biochemical signaling cascade of FAK, PKC and RhoA, which is associated with osteoclast differentiation. In addition, there is a close relationship between osteoclasts and osteoblasts. After RANKL from mechanical stimulated osteoblasts, the expression of LPL in preosteoclasts is increased, which is regulated by PI3K/AKT/SP1 pathway and promotes the formation of mature osteoclasts. STIM1 and TRPV4 act as mechanical transduction channels of osteoclasts in the early and late stages of differentiation.

All in all, mechanical forces trigger the growth and differentiation of osteoblasts. The short duration of fluid flow at physiological ranges is conducive to promoting the proliferation and survival of osteoblasts.^110^ Interestingly, lack of mechanical stimulation *in vivo* leads to osteoblast apoptosis and osteoporosis, while inappropriate high strain also results in substantial cell detachment and breakdown of cell adhesion, which is necessary for physiological resistance to high strain.^111^ Importantly, excessive pressure on osteoblasts induces the expression of extracellular antagonists of BMP, thereby inhibiting osteoblast formation.^39^ The continuous compression of osteoblasts *in vitro* induces osteoblasts to produce inflammatory cytokines and their receptors.^38^ This phenomenon is more desirable through the autocrine motion of IL-1β, which is accelerated via mechanical stress and may be associated to the signaling pathway of RANKL and its corresponding receptor RANK. In addition, continuous compression in osteoblasts *in vitro* induces the production of inflammatory cytokines and their receptors in osteoblasts, causing or aggravating OA. Molecular interactions between osteoblasts and osteoclasts have been documented during bone remodeling. Abnormal mechanical stress triggers osteoblast metabolic dysregulation, characterized by increased expression of PGE2, MMP-3, -9 and -13, and RANKL, as well as decreased OPG production and regulation of osteoclast metabolism^33^ (Fig. 3a).

### 4.3 Osteoclast perception of mechanical stimulation

The predominant characteristic of osteoclasts is to absorb bone, take part in bone reconstruction, and interact with osteoblasts to maintain bone stability. Under pathological conditions, osteoclasts are generally accountable for initiating normal bone remodeling and mediating bone loss by way of increasing absorptive activity.^112^ Sun *et al.* detected the Piezo1 activation and level of cationic current induced by mechanical stimulation on the cell membrane in osteoclast cell line RAW264.7.^113^ Emerging studies shows that osteoclasts respond to mechanical stimuli that affect cell differentiation, such as fluid shear stress, tension, compression force, microgravity, and vibration.^114^ However, the specific mechanism of osteoclast perception of mechanical stimulation remains unclear. Osteoclasts sense their mechanical environment and regulate the deposition or absorption of the bone matrix. It has been investigated that mechanical stretching promoted the bone resorption activity of osteoclasts by using a device that applies mechanical stretching to an ivory/plastic plate assembly in which cells can be cultured. Mature and highly enriched osteoclasts were cultured on the ivory/plate assembly for 2, 12 and 24 ​h while undergoing intermittent tensile strain. The expression levels of osteoclast marker enzyme, TRAP and CTSK mRNA in stretching osteoclasts were enhanced, and the formation of absorption pits was increased, indicating that mechanical stretching upregulated the bone absorption activity of osteoclasts. Stretch-activated cation (SA-cat) channel blockers considerably inhibited the upregulation of marker mRNA levels and pit formation after stretching for 24 ​h. These findings suggest that mature osteoclasts may respond to mechanical stretching related to the SA-cat channel, thereby upregulating their bone resorption activity.^115^

#### 4.3.1 Cytoskeleton-associated proteins

Osteoclast activation is initiated by adhesion to the bone surface, followed by cytoskeletal rearrangement, sealing zone formation, and polarized wrinkled membranes. Ca^2+^ channels stromal interaction molecule 1 (STIM1) and transient TRPV4 act as mechanical transduction channels of osteoclasts in the early and late stages of differentiation, respectively.^116^ Some scholars have established mechanical models of polydimethylsiloxane (PDMS) substrates, which simulate the physiological and mechanical properties of extracellular microenvironments with different cell culture rigidities. Harder PDMS substrates accelerate osteoclast differentiation with adjustments in morphology and fusion/fission activity and contribute to the upregulation of typical osteoclast markers NFATc1, Acp5, CTSK, MMP-9, TRAF6 and so on. In addition, activation of cytoskeletal-associated adhesion molecules (including fibronectin and integrin αvβ3) is detected on harder substrates, followed by biochemical signaling cascades of paxillin, FAK, PKC, and RhoA. It concludes that changes in the stage of mechanically sensitive cytoskeleton-related adhesion molecules in response to substrate stiffness might be associated to osteoclast differentiation. It has been demonstrated that the hardness of the extracellular substrate is a primary determinant of osteoclast differentiation and function. Higher stiffness upregulates osteoclast activity and differentiation, revealing the mechanical regulation of osteoclast activity in bone homeostasis and diseases.^117^ The osteoclast skeleton is a unique structure that polarizes cells’ absorption mechanism to the interface of osteoclast, where it creates an isolated reabsorption microenvironment consisting of actin rings surrounding the fold boundary.^118^ The actin cytoskeleton plays a unique role in osteoclast formation and activity. When osteoclasts are labeled with filamentous or F-actin fluorescent markers, podocytes can be visualized as dense dot-like structures surrounded by looser actin. The podosomes can sense surface hardness, topography and components based on the presence of matrix proteins that can participate in integrins.^114^^,^^119^, ^120^, ^121^ The various substructures that connect individual podosomes allow the induction and generation of mechanical forces on the plasma membrane. L-plastin (LPL) is an actin-binding protein essential for actin cytoskeletal tissue.^122^ LPL deletion obstructs preosteoclast fusion by inhibiting filopodia formation and increases the number of preosteoclasts, which release PDGF-BB to promote CD31^hi^Emcn^hi^ vascular growth and bone formation.^123^ In conclusion, LPL could regulate osteoclast fusion and affects the perception of mechanical signals.

#### 4.3.2 Mechanoresponsive extracellular vesicle formation

As a communicating mediator of biomolecules between cells, extracellular vehicles are significant in regulating homeostasis between a range of mechano-sensing tissues.^124^ In bone, osteoclasts as mechanically sensitive cells release large amounts of extracellular vehicles during bone remodeling and act on surrounding nerve endings, endothelial cells, osteoblasts to regulate their activity functions. The formation and secretion of vesicles depend on mechanoresponsive cytoskeletal recombination.^125^^,^^126^ Throughout bone remodeling, subchondral bone osteoclasts sense and respond to chronic mechanical stimuli. It has been noted that extracellular vesicles secreted by subchondral bone osteoclasts have existed in the abnormal mechanical environment of OA, initiating subchondral bone microenvironment disturbances and the progression of OA. Liu *et al.* identified a transfer of exosomal osteoclast-derived microRNAs contributes to OA progression during the progression of surgery-induced OA in mice.^127^ Blocking the secretion of osteoclasts-originated exosomes by short interfering RNA-mediated silencing of GTPase Rab27a (a cytoskeletal regulatory protein of intracellular transport) or systemic administration of a new osteoclasts-targeted exosome inhibitor substantially blunted the progression of OA. Mechanistically, the exosomal transfer of osteoclast-derived miR-214-3p to chondrocytes reduces the resistance of cartilage to matrix degeneration, osteochondral angiogenesis and sensory innervation in subchondral bone microenvironment (Fig. 3b).

## 5 Targeting mechanical homeostasis of subchondral bone for OA treatment

### 5.1 Maintaining proper mechanical stimulation via exercise therapy

Proper mechanical stimulation is essential for bone health and OA recovery. Patients with OA often give up exercise because of pain, whereas long-term immobility leads to loss of leg muscle strength and increased disability, as well as the faster progression of knee OA.^128^ International guidelines for the management of OA advise exercising as the core of non-drug therapy. Younger participants with knee OA who are not indicated for joint replacement may benefit more from exercise therapy.^129^ Although exercise can be used to prevent or treat OA, abnormal biomechanics and abnormal joint forces have been identified as potential culprits in the onset and progression of OA.^130^ Exercises regulate subchondral bone remodeling improvement in DMM-operated knees in a dose-dependent manner. Moderate exercises can increase BMP-2, BMP-4, BMP-6, BMP receptor 2, p-Smad5, and the inhibitor of DNA binding protein-1 expression in the superficial zone chondrocytes and suppress cartilage degeneration, osteophyte growth, subchondral bone damage, and osteoclast-mediated subchondral bone resorption.^131^ However, intense exercises have negative impact on BMP expression and even cause progression of OA.^130^ Personalized exercise prescriptions should be formulated according to the lesion severity and needs of OA patients, including resistance load, number of repetitions, exercise speed and weekly session frequency.

### 5.2 Restoring mechanical homeostasis through surgical treatment

In addition to conservative treatment, arthroplasty and osteotomy have been documented as clinically effective treatments. Joint alternative is limited to patients with moderate to severe OA.^132^ Osteotomy around the knee has been chosen as an alternative surgical treatment for younger patients who wish to retain more knee function at early stage of OA progression. The principle of knee osteotomy is to rebalance the forces between the medial and lateral compartments and to relieve the stress on the cartilage and subchondral bone through bone cutting and correcting the mechanical axis.^133^ Osteotomy techniques around the knee, including osteotomy, have been designed to redistribute forces in the interstitial compartment of the knee joint. This operation preserves the normal anatomy and allows the knee joint to recover well. Since osteotomy preserves the anatomical shape of the joint, it has the benefits of proprioception maintenance and speedy healing of joint useful efficacy, which radically delays the development of OA.^134^ It has unique advantages compared with knee replacement. The danger of contamination after knee alternative is high, and youthful sufferers who have passed through substitute may additionally want revision surgical treatment late. Osteotomy is relatively simple, low risk and results in rapid recovery in almost all cases. While the long-term effects remain uncertain, it is widely expected that this method will be improved in terms of accuracy, effectiveness and convenience with the advent of new technologies.

## 6 Conclusion

Mechanical stimulation is a key environmental factor in maintaining joint homeostasis and the development of OA. The response of mechanosensitive cells to mechanical forces in their environment impacts their behaviors and alteration of responses in other cells, highlighting the significance of mechanical cues in both cellular physiology and disease pathogenesis. Overwhelming literature substantiates that osteocytes, osteoblasts and osteoclasts respond to mechanical stimuli through various mechanosensory mechanisms and signaling pathways in subchondral bone. Little is currently known about whether a dominant pathway influences other signaling pathways, nor is it clear whether there is a signaling pathway most sensitive to stress stimulation. OA treatment and repair of subchondral bone remodeling need in-depth research based on consideration to biomechanical functional characteristics and mechanobiology of subchondral bone. The influence of quantitative mechanical conditions on subchondral bone cells needs further study *in vitro* and *in vivo*.

## Ethics approval and consent to participate

Not applicable.

## Author contributions

HWH, RF, and SWD conceived the study, coordinated it, and helped draft the manuscript. FK, XY, and YW drafted the manuscript and substantively revised it. HWH, RF, and FK designed the figures. XL, JL, and JZ helped revise the figures. SWD contributed to manuscript editing. FK, XY, and YW helped revise the manuscript. All authors read and approved the final manuscript.

## Declaration of competing interest

The authors declare that they have no known competing financial interests or personal relationships that could have appeared to influence the work reported in this paper.
